# Is time‐restricted eating (8/16) beneficial for body weight and metabolism of obese and overweight adults? A systematic review and meta‐analysis of randomized controlled trials

**DOI:** 10.1002/fsn3.3194

**Published:** 2022-12-19

**Authors:** Lu Huang, Yan Chen, Shu Wen, Danhua Lu, Xiaoyang Shen, Hongxia Deng, Liangzhi Xu

**Affiliations:** ^1^ Department of Obstetrics and Gynecology, West China Second University Hospital Sichuan University Chengdu China; ^2^ Reproductive Endocrinology and Regulation Laboratory, West China Second University Hospital Sichuan University Chengdu China; ^3^ Key Laboratory of Birth Defects and Related Diseases of Women and Children (Sichuan University) Ministry of Education Chengdu China; ^4^ The Joint Laboratory for Reproductive Medicine of Sichuan University‐The Chinese University of Hong Kong Chengdu China; ^5^ Department of Critical Care Medicine West China Hospital of Sichuan University Chengdu China

**Keywords:** body weight, meta‐analysis, metabolism, systematic review, time‐restricted eating

## Abstract

Time‐restricted eating (TRE) is a new therapeutic strategy for the management of weight loss and dysmetabolic diseases. At present, TRE (8/16, 8 h eating:16 h fasting) is the most common form of TRE. Therefore, this meta‐analysis included randomized controlled trials (RCTs) on TRE (8/16) in overweight and obese adults to determine its impact on body weight and metabolism. Articles reviewed from PubMed, Ovid MEDLINE, Embase, and Cochrane Central Register for the relevant RCTs that compared TRE (8/16) to non‐TRE in overweight and obese adults. Eight RCTs were included in this meta‐analysis. Participants following TRE (8/16) showed significant body weight reduction (mean difference [MD]: −1.48 kg, 95% confidence interval [CI]: −2.53 to −0.44) and fat mass reduction (MD: −1.09 kg, 95% CI: −1.55 to −0.63). There was no significant difference in lean mass change with TRE intervention (MD: −0.48 kg, 95% CI: −1.02 to 0.05, *p* = .08, *I* ^2^ = 41%). The energy restriction and early TRE (eTRE) subgroups resulted in greater weight loss. TRE (8/16) showed beneficial effects on the homeostatic model assessment of insulin resistance (HOMA‐IR, MD: −0.32, 95% CI: −0.59 to −0.06), but had no significant effect on other parameters of glucose metabolism and lipid profiles. In conclusion, TRE (8/16), especially eTRE, or in combination with caloric intake restriction, is a potential therapeutic strategy for weight control in overweight and obese adults. TRE (8/16) also reduced HOMA‐IR; therefore, it may have a positive effect on glucose metabolism.

## Graphical abstract

After analysis, We believe Time‐restricted eating (TRE,8/16) is a potential therapeutic strategy for controlling weight in adults with overweight and obesity, especially the elderly and people who do not exercise regularly, and eTRE maybe the better choice for weight loss. TRE (8/16) also reduces HOMA‐IR, so maybe it has a positive effect on glucose metabolism. This review aimed to assess the time‐restricted eating (8/16) on body weight and metabolism in overweight and obese adults. A meta‐analysis and systematic review was conducted up to May 2022.

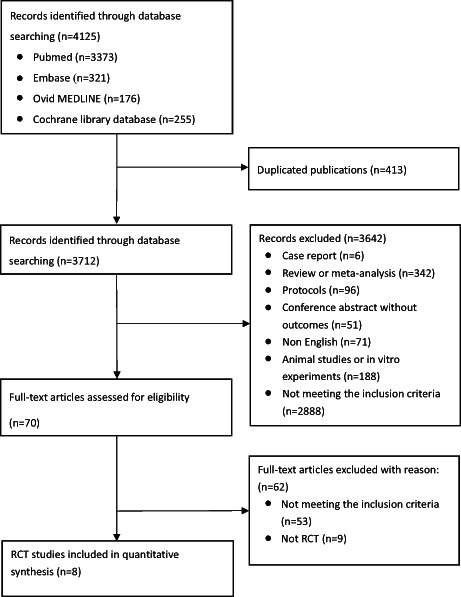

## 1 INTRODUCTION

Obesity represents a pathological state caused by multiple factors, such as a diet rich in sugar and oil and poor living habits. Obesity is associated with the development of type 2 diabetes mellitus (T2DM), cardiovascular diseases, some types of cancer, and other adverse pathological conditions (Williams et al., 2015). Furthermore, novel data indicate that obesity and impaired metabolic health are important risk factors for severe coronavirus disease 2019 (Stefan, 2022; Stefan et al., 2021). Obesity has a high global prevalence, and it is not an exaggeration to call it an epidemic (Aguilar‐Gallardo et al., 2022). A study by Ward et al. (2019) suggested that by 2030, nearly one in every two US adults will have obesity, and severe obesity is estimated to be found in close to 25% of US adults. In China, standardized mean body mass index (BMI) levels rose from 22.7 kg/m^2^ in 2004 to 24.4 kg/m^2^ in 2018 and obesity prevalence from 3.1% to 8.1% (Wang et al., 2021). Effectively curbing obesity is a pressing issue worldwide. Lifestyle interventions, including reduced energy intake and increased regular exercise, have been the first‐line therapies in efforts to combat obesity (Anderson et al., 2001). However, many individuals find it difficult to adhere to calorie restriction (CR) diets (Del Corral et al., 2009). Therefore, adherence to dietary strategies to achieve satisfactory weight loss and metabolic benefits remains prevalent.

Time‐restricted eating (TRE) is a type of intermittent fasting (IF) in which eating is restricted to a reduced number of fixed hours per day, prolonging the fasting period (Chaix et al., 2019; de Cabo & Mattson, 2019; Panda, 2016; Zarrinpar et al., 2016). Studies in rodents have demonstrated that TRE reduces body weight and improves markers of metabolic health without altering energy consumption (Hatori et al., 2012; Sherman et al., 2012). However, the results of human clinical trials on TRE are controversial, especially regarding weight loss, lipid profiles, and glucose metabolism. Therefore, it is necessary to explore whether TRE is beneficial to body weight and metabolism in overweight and obese adults.

Systematic reviews and meta‐analyses published earlier point to a couple of the health benefits of TRE (Adafer et al., 2020; Chen et al., 2021; Moon et al., 2020; Pellegrini et al., 2020; Pureza et al., 2021). However, a closer examination of these meta‐analyses reveals some limitations. First, TRE strategies are not standardized, with fasting windows between 12 and 20 h (Adafer et al., 2020; Chen et al., 2021; Moon et al., 2020; Pellegrini et al., 2020; Pureza et al., 2021). Second, the duration of the intervention varies from a few days to months (Adafer et al., 2020; Chen et al., 2021; Moon et al., 2020; Pellegrini et al., 2020; Pureza et al., 2021). Finally, the types of studies included were inconsistent, including randomized controlled trials (RCTs) and observational studies (Adafer et al., 2020; Moon et al., 2020; Pellegrini et al., 2020; Pureza et al., 2021). All these reasons can lead to high heterogeneity among studies. In addition, a number of new RCTs have emerged in recent years with inconsistent results. Therefore, a meta‐analysis that will limit the above heterogeneity and include all relevant TRE RCTs available thus far is necessary to evaluate its impact on health. At present, TRE (8/16, 8 h eating:16 h fasting) is considered the most common form of TRE (Adafer et al., 2020). Thus, this article reviews currently available RCTs on TRE (8/16) in overweight and obese adults to determine its impact on body weight and metabolism.

## 2 METHODS

This meta‐analysis was registered at https://www.crd.york.ac.uk/prospero/ with registration number CRD42022335421 on June 5, 2022. After registration, the analysis was performed at West China Second University Hospital, Sichuan University, according to the recommendations from the Cochrane Handbook for Systematic Reviews, as well as the Preferred Reporting Items for Systematic Reviews and Meta‐Analyses statement guidelines.

### 2.1 Retrieval strategy

Two authors (HL and CY) separately searched PubMed, Ovid MEDLINE, Embase, and Cochrane Central Register for relevant RCTs up to May 6, 2022. The search strategy was based on database‐specific subject headings and keywords. Medical subject headings (MeSH) and free‐text search terms were used. Restrictions on human studies were also included. We also manually scanned the reference lists from trials, review articles, and reports to identify any other relevant data. Search terms included combinations of “time‐restricted feeding,”, “time‐restricted diet,” “time‐restricted meal,” “time‐restricted fasting,” or “time‐restricted eating” and “body mass index,” “adiposity,” “overweight,” or “obesity” (the details of the search strategy are presented in Appendix S1).

### 2.2 Inclusion criteria

The following were the inclusion criteria. (1) Population: adults aged 18 years or older; overweight or obese people with BMI ≥25 kg/m^2^. (2) Intervention: at least one treatment arm with TRE (8/16) intervention. (3) Comparison: non‐TRE (unlimited eating time, regular eating schedule, or usual eating habits). (4) Study duration≥4 weeks. (5) Study type: RCT.

### 2.3 Exclusion criteria

Studies with ambiguous descriptions of the TRE regimen; conference abstracts without outcomes, reviews, case reports, or protocols; studies including participants with acute or chronic diseases, such as gastrointestinal diseases or cancer, that affect the outcomes; or non‐RCTs and nonrelevant studies were the exclusion criteria.

### 2.4 Outcomes

The primary outcome was the change in body weight between the TRE (8/16) and control groups. Secondary outcomes were the changes in body composition (lean mass, fat mass, and visceral fat mass), waist and hip circumference, blood pressure, lipid profiles (high‐density lipoprotein cholesterol [HDL‐C], low‐density lipoprotein cholesterol [LDL‐C], total cholesterol [TC], and triglycerides [TG]), and glucose metabolism (blood glucose, hemoglobin A1c [HbA1c], homeostatic model assessment of insulin resistance [HOMA‐IR], and insulin) between the TRE (8/16) and control groups. The included studies were required to assess one of the primary or secondary outcomes.

### 2.5 Data extraction

Based on the inclusion and exclusion criteria, two authors (HL and CY) sequentially enrolled the trials and extracted data independently into a predesigned database. The following information was extracted: publication information (first author's name and publication year), participant characteristics (patients' age, sample size, and BMI), and primary and secondary outcomes. Discrepancies in eligibility between the two researchers were resolved through discussions. If necessary, a third researcher (XLZ) was involved.

Continuous variables were expressed as mean ± standard deviation (SD). For the trials that merely reported the range/interquartile range or for which data were expressed with figures, the formula introduced by Wan et al. (2014) and the program Engauge Digitizer 5.1 (M. Mitchell, Engauge Digitizer, http://digitizer.sourceforge.net) were used to estimate or extract data.

### 2.6 Bias assessment

The risk of bias was assessed according to the Cochrane tool for assessing the risk of bias in randomized clinical trials (Table S1), including the following domains: sequence generation, concealment of allocation, blinding, incomplete outcome data addressed, selective outcome reporting, and others.

### 2.7 Statistical analysis

Data were analyzed using RevMan version 5.3.0 (Cochrane Collaboration, DerSimonian & Laird, 1986). The pooled effect sizes are presented as mean differences (MD) and 95% confidence intervals (CI) using the mean with SD values before and after the TRE intervention. In the case of unavailable SD, it was obtained from standard errors and confidence intervals for group means based on the approach described in the Cochrane Handbook for Systematic Reviews of Intervention (Higgins et al., 2019). Heterogeneity between the pooled studies was represented by *I* ^2^ value. *I* ^2^ ≥ 50% was defined as high heterogeneity. The Hartung–Knapp–Sidik–Jonkman (HKSJ) method (IntHout et al., 2014; Röver et al., 2015) was further used for the pooled data with high heterogeneity or a limited number of studies (<5 trials). A fixed effects model was used if the result showed *I* ^2^ < 50%; otherwise, a random effects model was used. Sensitivity analysis was performed to assess the robustness and reliability of the pooled results.

## 3 RESULTS

### 3.1 Literature search

**FIGURE 1:**
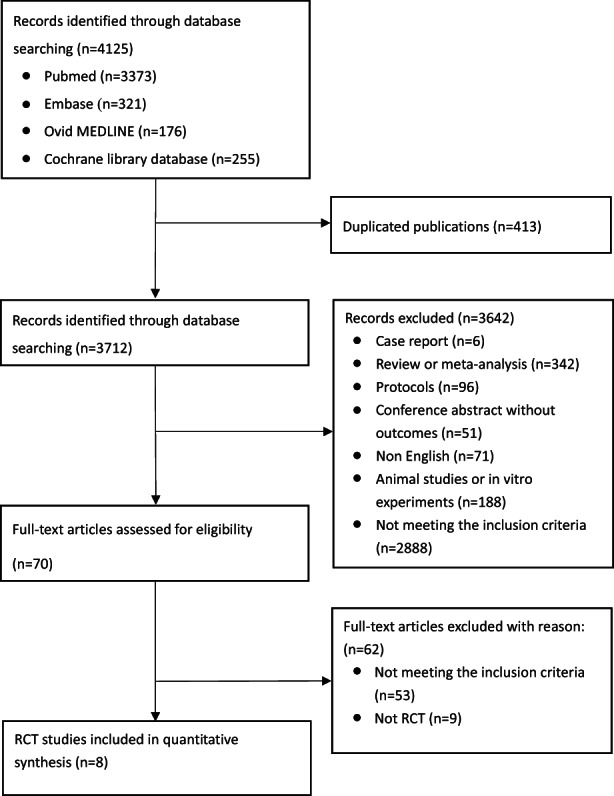
Flow diagram of selecting process. RCT, randomized controlled trial.

A total of 4125 related articles (PubMed 3373, Embase 321, Ovid MEDLINE 176, Cochrane Library database 255) were identified by the original screening (Figure 1). After removing 413 duplicate studies, 3712 studies were carefully screened based on their titles and abstracts. According to the exclusion criteria, 342 reviews or meta‐analyses, 96 protocols, 51 conference abstracts without outcomes, 71 non‐English studies, 188 animal studies or in vitro experiments, and 6 case reports were excluded. Another 2888 were excluded because they did not meet the inclusion criteria. Through careful screening of the full text of the articles, 62 records were excluded for various reasons: TRE not meeting 8:16 in 25 studies, BMI < 25 or BMI not mentioned in 11 studies, other drugs used in 17 studies, and nine non‐RCTs. Finally, eight studies of 464 patients were included in the data analysis (Amodio et al., 2016; Chow et al., 2020; Domaszewski et al., 2020; Isenmann et al., 2021; Kotarsky et al., 2021; Kunduraci & Ozbek, 2020; Liu et al., 2022; Lowe et al., 2020). The main characteristics of the eight eligible studies are presented in Table 1.

**TABLE 1:** The characteristic of included articles

Studies	Sample size (*n*; M/F)		Participants					Duration	Intervention strategies		Outcomes
	TRE	Control	Characteristics	Age (TRE)	Age (control)	BMI (TRE)	BMI (control)		TRE	Control	
Amodio et al. (2016)	12 (0/12)	11 (0/11)	Menopausal women with metabolic syndrome	55.6 ± 5	55.3 ± 5	29.3 ± 4.9	29 ± 1.2	45 days	8 h eating (7 am–3 pm); 1600 kcal/day	Unlimited eating time; 1600 kcal/day	a, f, k, l
Chow et al. (2020)	11 (2/9)	9 (1/8)	Overweight or obesity participants	46.5 ± 12.4	44.2 ± 12.3	33.8 ± 7.6	34.4 ± 7.8	12 weeks	Self‐selected 8‐h eating window for ad libitum intake	Eating ad libitum per their usual habits	a, b, c, e, h, i, k, l, m, n, o
Domaszewski et al. (2020)	25 (0/25)	20 (0/20)	Nonsmoking women over 60 years, belonged to a seniors' association	65 ± 4	66 ± 4.7	28.99 ± 5.18	26.99 ± 4.20	6 weeks	8 h eating (12 pm–8 pm)	Eating ad libitum based on their previous habits	a, c
Isenmann et al. (2021)	18 (8/10)	17 (6/11)	Regularly physically active participants	27.9 ± 5.3	27.4 ± 5.8	26.3 ± 3.0	25.7 ± 3.3	8 weeks	8 h eating (12 pm–8 pm), 45%–65% of the total energy intake should be from carbohydrates, 20%–35% from fats, and 20%–35% from proteins	No eating time restriction; following a healthy and balanced diet	a, b, c, d, f, g
Kotarsky et al. (2021)	11 (2/9)	10 (1/9)	Physically inactive people	45 ± 3	44 ± 2	29.4 ± 0.8	29.8 ± 0.8	8 weeks	8 h eating (12 pm–8 pm)	Eating ad libitum based on the regular eating schedule	a, b, c, d, f, g, h, j, m, o
Kunduraci and Ozbek (2020)	32 (16/16)	33 (15/18)	Diagnosed metabolic syndrome patients	47.44 ± 2.17	48.76 ± 2.13	36.58 ± 0.93	32.82 ± 0.72	12 weeks	8 h eating (8 am–4 pm, 9 am–5 pm, 10 am–6 pm, 11 am–7 pm); 25% energy restriction	No eating time restriction, 25% energy restriction	a, c, e, f, h, i, j, k, l, m, n, o
Liu et al. (2022)	69 (36/33)	70 (35/35)	Overweight or obesity participants	31.6 ± 9.3	32.2 ± 8.8	31.8 ± 2.9	31.3 ± 2.6	12 months	8 h eating (8 am–4 pm); 25% energy restriction	No eating time restriction, 25% energy restriction	a, b, c, e, f, h, i, j, k, l, n, o
Lowe et al. (2020)	59 (35/24)	57 (35/22)	Overweight or obesity participants	46.8 ± 10.8	46.1 ± 10.3	32.7 ± 4.2	32.6 ± 3.4	12 weeks	8 h eating (12 pm–8 pm)	Standard three‐meals‐per‐day diet; snacking between meals was permitted	a, b, c, d, e, f, g, h, i, j, k, l, m, n, o

*Note*: a, changes in body weight; b, changes of body lean mass; c, changes of body fat mass; d, changes of visceral fat mass; e, changes of blood pressure; f, changes of waist circumference; g, changes of hip circumference; h, changes of high‐density lipoprotein cholesterol (HDL‐C); i, changes of low‐density lipoprotein cholesterol (LDL‐C); j, changes of total cholesterol; k, changes of total triglycerides; l, changes of blood glucose; m, changes of HbA1c; n, changes of HOMA‐IR; o, changes of insulin.

Abbreviations: BMI, body mass index; F, female; M, male; TRE, time‐ restricted eating.

### 3.2 Risk of bias

**FIGURE 2:**
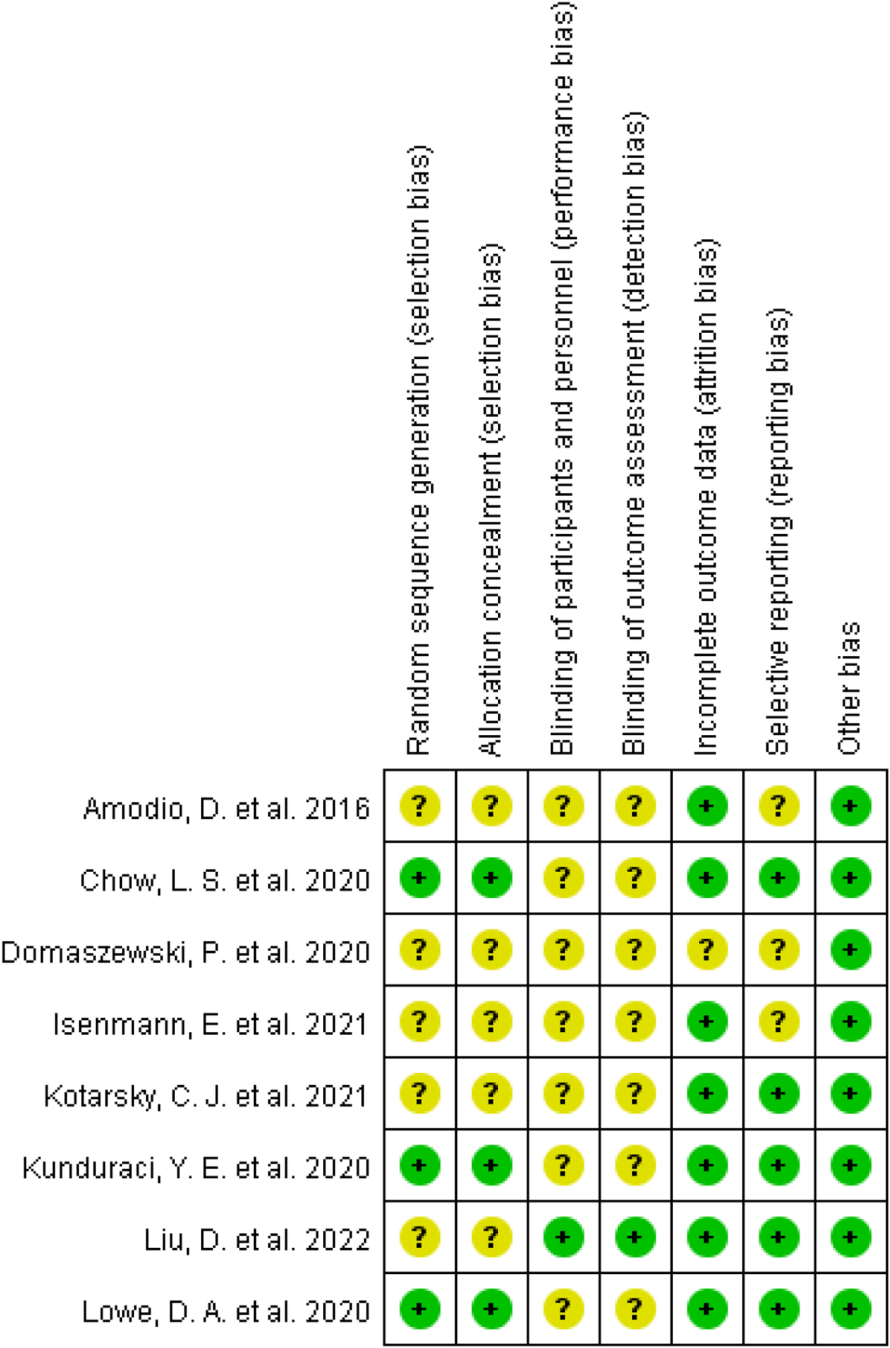
Risk of bias summary. Risk of bias item for each included RCT according to Cochrane Risk‐of‐Bias tool. +, low risk; ?, unclear risk.

The risk of bias of the studies is shown in Figure 2, Table S1 and Figure S1. Three studies (Chow et al., 2020; Kunduraci & Ozbek, 2020; Lowe et al., 2020) presented a specific random assortment method. The blinding of observers (trial personnel) was described in one study (Liu et al., 2022). One study did not report the number of dropouts (Domaszewski et al., 2020), and other studies had a low dropout rate.

### 3.3 Outcomes

#### 3.3.1 The primary outcome

##### Effect of TRE on body weight

**FIGURE 3:**
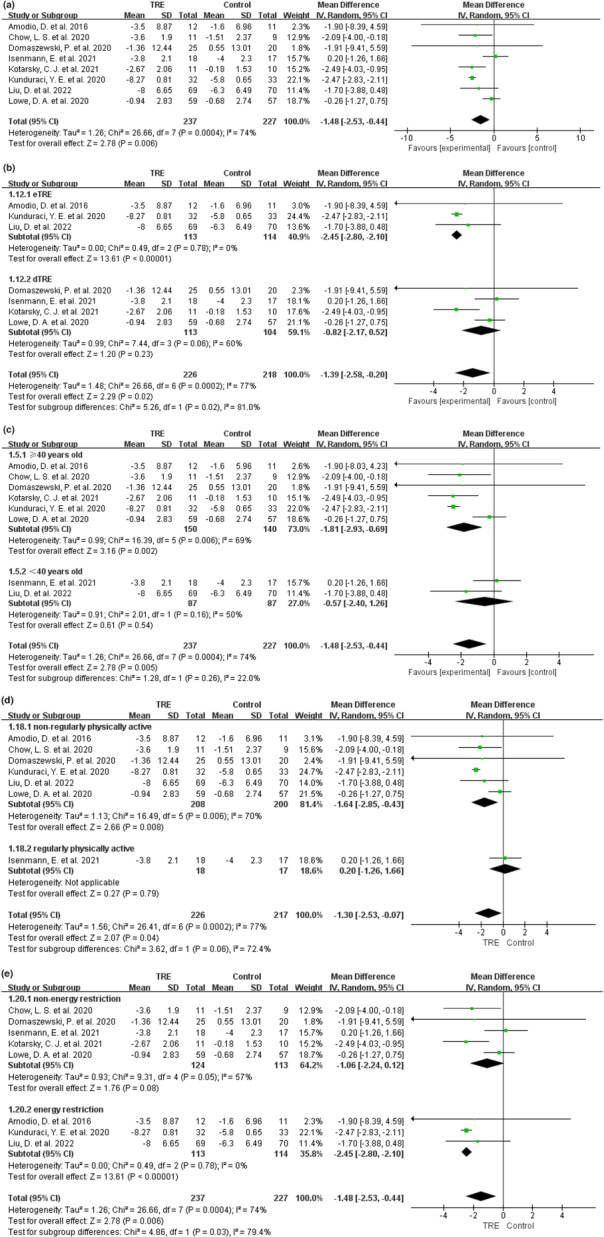
Effect of TRE versus controls on body weight loss. (a) Effect of TRE versus controls on body weight loss of all studies. The effect on the body weight loss of TRE versus controls following subgroup assignment based on (b) fasting windows, (c) participants’ ages (≥40 years old vs. <40 years old), (d) different amount of exercise (nonregularly physically active participants vs. regularly physically active participants), and (e) whether to limit energy intake.

All eight studies (Amodio et al., 2016; Chow et al., 2020; Domaszewski et al., 2020; Isenmann et al., 2021; Kotarsky et al., 2021; Kunduraci & Ozbek, 2020; Liu et al., 2022; Lowe et al., 2020), including 464 participants, evaluated the changes in body weight. All comparisons were made with the control groups, unless otherwise stated. The MD of weight change using a random effects model was −1.48 kg (95% CI: −2.53 to −0.44, *p* = .006, *I* ^2^ = 74%), indicating a significant weight loss using the TRE intervention (Figure 3a). Removing the study by Kunduraci and Ozbek (2020), based on the leave‐one‐out approach, reversed the heterogeneity, and maintained a significant weight reduction in the TRE group (MD: −1.13 kg, 95% CI: −2.08 to −0.18; *p* = .02; *I* ^2^ = 40%; Figure S2). This trend was also observed in the left‐out study.

In the subgroup analysis, fasting windows were divided into early TRE (eTRE) and delayed TRE (dTRE). Specifically, the food intake of eTRE started before 12:00 pm and the food intake of dTRE started at 12:00 pm or later. The fasting window data of Chow et al. (2020) could not be obtained, so we analyzed the data of other articles. The subgroup of eTRE (Amodio et al., 2016; Kunduraci & Ozbek, 2020; Liu et al., 2022) reported a significant weight reduction (MD: −2.45 kg, 95% CI: −2.80 to −2.10; *p* < .00001; *I* ^2^ = 0%), while the dTRE subgroup (Domaszewski et al., 2020; Isenmann et al., 2021; Kotarsky et al., 2021; Lowe et al., 2020) demonstrated no significant effect (MD: −0.82 kg, 95% CI: −2.17 to 0.52; *p* = .23; *I* ^2^ = 60%; Figure 3b). Because of the data pooled with high heterogeneity (*I* ^2^ = 60%) and a limited number of studies (four studies) in the dTRE group, the HKSJ method was further conducted, but it did not alter the result (standard mean difference [SMD] = −0.24, 95% CI: −1.06 to 0.58, *p* = .42; Figure S3a).

In subgroup analysis according to ages of participants, the ≥40 years old subgroup (Amodio et al., 2016; Chow et al., 2020; Domaszewski et al., 2020; Kotarsky et al., 2021; Kunduraci & Ozbek, 2020; Lowe et al., 2020) reported a significant weight reduction with TRE intervention (MD: −1.81 kg, 95% CI: −2.93 to −0.69; *p* = .002; *I* ^2^ = 69%), while the <40 years old subgroup (Isenmann et al., 2021; Liu et al., 2022) demonstrated no significant effect (MD: −0.57 kg, 95% CI: −2.40 to 1.26; *p* = .54; *I* ^2^ = 50%; Figure 3c). Because of the data pooled with high heterogeneity in the ≥40 years subgroup, removing a study by Kunduraci and Ozbek (2020), based on the leave‐one‐out approach, reversed the heterogeneity, and maintained the significant weight reduction in the TRE group (MD: −1.46 kg, 95% CI: −2.71 to −0.22; *p* = .02; *I* ^2^ = 42%; Figure S3b). The same trend was observed in the left‐out study.

In the subgroup analysis according to different exercises, the definition of regular physical activity was that the participants exercised continuously at least twice a week for 6 months, at recommended exercise intensity and amounts during the study, and their completions were monitored. Nonregular physical activity was defined as nondeliberate exercise with no recommendation for physical activity during the study. The nonregularly physically active subgroup (Amodio et al., 2016; Chow et al., 2020; Domaszewski et al., 2020; Kunduraci & Ozbek, 2020; Liu et al., 2022; Lowe et al., 2020) reported a significant weight reduction with TRE intervention (MD: −1.64 kg, 95% CI: −2.85 to −0.43; *p* = .008; *I* ^2^ = 70%). Because there was only one study (Isenmann et al., 2021) in the regular physically active subgroup, pooled data analysis could not be conducted. In this study (Isenmann et al., 2021), the regularly physically active group demonstrated no significant effect on weight reduction with TRE intervention (MD: 0.2 kg, 95% CI: −1.26 to 1.66; *p* = .79; Figure 3d). Because of the high heterogeneity in the nonregularly physically active subgroup, removing a study by Kunduraci and Ozbek (2020) based on the leave‐one‐out approach reversed the heterogeneity and maintained a significant weight reduction in the TRE group (MD: −0.84 kg, 95% CI: −1.66 to −0.02; *p* = .04; *I* ^2^ = 0%; Figure S3c). The same trend was observed in the left‐out study.

In three studies (Amodio et al., 2016; Kunduraci & Ozbek, 2020; Liu et al., 2022), interventions included the same energy restriction in both groups in addition to TRE; therefore, we performed a subgroup analysis. The energy restriction subgroup (Amodio et al., 2016; Kunduraci & Ozbek, 2020; Liu et al., 2022) showed a significant weight reduction with TRE intervention (MD: −2.45 kg, 95% CI: −2.80 to −2.10; *p* < .00001; *I* ^2^ = 0%), while the nonenergy restriction subgroup (Chow et al., 2020; Domaszewski et al., 2020; Isenmann et al., 2021; Kotarsky et al., 2021; Lowe et al., 2020) demonstrated no significant effect (MD: −1.06 kg, 95% CI: −2.24 to 0.12; *p* = .08; *I* ^2^ = 57%; Figure 3e). Because of the data pooled with high heterogeneity (*I* ^2^ = 57%) in the nonenergy restriction group, the HKSJ method was further conducted, but it did not alter the results (SMD = −0.34, 95% CI: −1.02 to 0.34, *p* = .23; Figure S3d).

#### 3.3.2 The secondary outcomes

##### Effect of TRE on body composition

The TRE intervention significantly reduced fat mass (MD: −1.10 kg, 95% CI: −1.51 to −0.68, *p* < .00001, *I* ^2^ = 13%; Figure S4a). In subgroup analysis according to fasting windows, both the eTRE and the dTRE decreased the body fat mass (MD: −1.42 kg, 95% CI: −2.13 to −0.71, *p* < .0001, *I* ^2^ = 0%, and MD: −0.96 kg, 95% CI: −1.53 to −0.40, *p =* .0008, *I* ^2^ = 45%, respectively; Figure S4b). In subgroup analysis, the nonregularly physically active subgroup (Chow et al., 2020; Domaszewski et al., 2020; Kunduraci & Ozbek, 2020; Liu et al., 2022; Lowe et al., 2020) reported a significant fat mass reduction with TRE intervention (MD: −1.01 kg, 95% CI: −1.51 to −0.51; *p* < .0001; *I* ^2^ = 0%). Because there was only one study (Isenmann et al., 2021) in the regular physically active subgroup, no pooled analysis was possible. In the study (Isenmann et al., 2021), the regularly physically active subgroup demonstrated no significant effect on fat mass reduction with TRE intervention (MD: −0.5 kg, 95% CI: −1.67 to 0.67; *p* = .4; Figure S4c). In subgroup analysis according to age of participants, the ≥40 years subgroup (Chow et al., 2020; Domaszewski et al., 2020; Kunduraci & Ozbek, 2020; Liu et al., 2022; Lowe et al., 2020) reported a significant fat mass reduction with TRE intervention (MD: −1.17 kg, 95% CI: −1.63 to −0.70; *p* = .0001; *I* ^2^ = 29%), while the <40 years subgroup (Isenmann et al., 2021; Liu et al., 2022) demonstrated no significant effect with TRE intervention (MD: −0.81 kg, 95% CI: −1.75 to 0.14; *p* = .10; *I* ^2^ = 0%; Figure S4e). In subgroup analysis according to limit energy intake or not, both the nonenergy restriction and energy restriction subgroup decrease the body fat mass (MD: −0.92 kg, 95% CI: −1.44 to −0.40, *p* = .0005, *I* ^2^ = 29%, and MD: −1.42 kg, 95% CI: −2.13 to −0.71, *p* < .0001, *I* ^2^ = 0%, respectively; Figure S4f).

Five studies (Chow et al., 2020; Isenmann et al., 2021; Kotarsky et al., 2021; Liu et al., 2022; Lowe et al., 2020; 261 participants) evaluated changes in lean body mass. There was no significant difference in lean mass change with TRE intervention (MD: −0.48 kg, 95% CI: −1.02 to 0.05, *p* = .08, *I* ^2^ = 41%; Figure S4g). In the subgroup analysis, there were no differences in eTRE and dTRE in terms of changes in body lean mass (Figure S4h). In subgroup analysis, the nonregularly physically active subgroup (Chow et al., 2020; Liu et al., 2022; Lowe et al., 2020) reported a significant lean mass reduction with TRE intervention (MD: −0.71 kg, 95% CI: −1.27 to −0.16, *p* = .01, *I* ^2^ = 15%). Because there was only one study (Isenmann et al., 2021) in the regular physically active subgroup, no pooled analysis was possible. In this study (Isenmann et al., 2021), the regularly physically active subgroup demonstrated no significant effect on lean mass reduction with TRE intervention (MD: 0.35 kg, 95% CI: −0.50 to 1.20; *p* = .42; Figure S4i). In subgroup analysis according to age of participants, the ≥40 years group (Chow et al., 2020; Kotarsky et al., 2021; Liu et al., 2022) reported a significant lean mass reduction with TRE intervention (MD: −0.88 kg, 95% CI: −1.44 to −0.32; *p* = .002; *I* ^2^ = 0%), while the <40 years group (Isenmann et al., 2021; Liu et al., 2022) demonstrated no significant effect (MD: 0.01 kg, 95% CI: −0.63 to 0.64; *p* = .98; *I* ^2^ = 16%; Figure S4j). In subgroup analysis according to limited energy intake or not, both the nonenergy restriction and energy restriction subgroup showed no significant effect in lean body mass with TRE intervention (SMD: −0.35, 95% CI: −0.72 to 0.01, *p* = .06, *I* ^2^ = 47%, and SMD: −0.12, 95% CI: −0.46 to 0.21, *p* = .46, respectively; Figure S4k).

Three studies (Chow et al., 2020; Kotarsky et al., 2021; Lowe et al., 2020) including 87 participants evaluated the changes in visceral fat mass. The MD of visceral fat mass using the random effects model was −0.07 kg (95% CI: −0.17 to 0.04, *p* = .21, *I* ^2^ = 63%), indicating no significant difference in visceral fat mass change with TRE intervention (Figure S4l). Because of the data pooled with high heterogeneity (*I* ^2^ = 63%) and limited number of studies (three studies), the HKSJ method was further conducted, but it did not alter the result (SMD = −0.4, 95% CI: −1.32 to 0.52, *p* = .2; Figure S4l).

##### Effect of TRE on waist circumference and hip circumference

Changes in waist circumference were reported in six studies (Amodio et al., 2016; Isenmann et al., 2021; Kotarsky et al., 2021; Kunduraci & Ozbek, 2020; Liu et al., 2022; Lowe et al., 2020), and pooled data showed that there was significant waist circumference reduction with TRE intervention (MD: −1.62 cm, 95% CI: −1.88 to −1.36, *p* < .00001, *I* ^2^ = 26%; Figure S5a).

Three studies (Isenmann et al., 2021; Kotarsky et al., 2021; Lowe et al., 2020) including 102 participants evaluated the changes in hip circumference. The MD of hip circumference using the fixed effects model was −0.46 cm (95% CI: −1.44 to 0.53, *p* = .36, *I* ^2^ = 34%), indicating no significant difference in hip circumference changes with TRE intervention (Figure S5b).

##### Effect of TRE on blood pressure

Four studies (Chow et al., 2020; Kunduraci & Ozbek, 2020; Liu et al., 2022; Lowe et al., 2020) including 270 participants evaluated changes in blood pressure. The pooled data showed that there were no significant differences of systolic blood pressure and diastolic blood pressure changes with TRE intervention (MD: 2.36 mmHg, 95% CI: −1.78 to 6.51, *p* = .26, *I* ^2^ = 78%, and MD: 0.84 mmHg, 95% CI: −2.67 to 4.34, *p* = .64, *I* ^2^ = 79%, respectively; Figure S6). Because of the data pooled with high heterogeneity (*I* ^2^ ≥ 50%) and a limited number of studies (four studies), the HKSJ method was further conducted, but it did not alter the results (SMD = 0.61, 95% CI: −1.27 to 2.49, *p* = .38, and SMD = 0.58, 95% CI: −2.02 to 2.64, *p* = .44, respectively).

##### Effect of TRE on lipid profiles

There were no significant changes in HDL‐C levels with TRE intervention (MD: 0.75 mg/dl, 95% CI: −0.13 to 1.63, *p* = .09, *I* ^2^ = 0%; Figure S7a). Four studies (Chow et al., 2020; Kunduraci & Ozbek, 2020; Liu et al., 2022; Lowe et al., 2020) including 266 participants, evaluated changes in LDL‐C levels. The MD of LDL‐C change using fixed‐effects model was −0.66 mg/dl (95% CI: −3.06 to 1.74, *p* = .59, *I* ^2^ = 0%), indicating no significant difference of LDL‐C change with TRE intervention (Figure S7b). No significant differences were observed in the changes in TC between the TRE and control regimens (MD: 0.26 mg/dl, 95% CI: −3.03 to 3.55, *p* = .88, *I* ^2^ = 0%; Figure S7c). Five studies (Amodio et al., 2016; Chow et al., 2020; Kunduraci & Ozbek, 2020; Liu et al., 2022; Lowe et al., 2020) including 289 participants, evaluated the changes in TG. The MD of TG change using the fixed effects model was −7.13 mg/dl (95% CI: −15.85 to 1.59, *p* = .33, *I* ^2^ = 13%), indicating no significant difference in TG change with TRE intervention (Figure S7d).

##### Effect of TRE on glucose metabolism

Five studies (Amodio et al., 2016; Chow et al., 2020; Kunduraci & Ozbek, 2020; Liu et al., 2022; Lowe et al., 2020), including 293 participants, evaluated changes in blood glucose levels. There were no significant differences in blood glucose between the TRE and control group (MD: −1.78 mg/dl, 95% CI: −3.76 to 0.20, *p* = .08, *I* ^2^ = 0%; Figure S8a). Four studies (Chow et al., 2020; Kotarsky et al., 2021; Kunduraci & Ozbek, 2020; Lowe et al., 2020), including 149 participants, evaluated the changes in HbA1c. The MD of HbA1c change using the fixed effects model was −0.02% (95% CI: −0.07 to 0.04, *p* = .61, *I* ^2^ = 0%), indicating no significant difference in HbA1c change with the TRE intervention (Figure S8b). Five studies (Chow et al., 2020; Kotarsky et al., 2021; Kunduraci & Ozbek, 2020; Liu et al., 2022; Lowe et al., 2020), including 287 patients, reported changes in insulin levels. The combined data showed that there were no statistical differences in the changes in insulin between the TRE and the control group (MD: 0.07mIU/L, 95% CI: −0.80 to 0.93, *p* = .88, *I* ^2^ = 0%; Figure S8c). Four studies (Chow et al., 2020; Kunduraci & Ozbek, 2020; Liu et al., 2022; Lowe et al., 2020), including 269 patients, evaluated the changes in HOMA‐IR. The combined data showed a significant reduction in HOMA‐IR in the TRE group (MD: −0.32, 95% CI: −0.59 to −0.06, *p* = .02, *I* ^2^ = 0%; Figure S8d).

## 4 DISCUSSION

This meta‐analysis included eight eligible articles (Amodio et al., 2016; Chow et al., 2020; Domaszewski et al., 2020; Isenmann et al., 2021; Kotarsky et al., 2021; Kunduraci & Ozbek, 2020; Liu et al., 2022; Lowe et al., 2020) to evaluate the effects of TRE (8/16) on body weight and metabolism in overweight and obese adults. The combined data showed that TRE (8/16) reduced the body weight, fat mass, and waist circumference. However, there were no differences in the changes in lean mass and visceral fat between the TRE (8/16) and control groups. In addition, TRE (8/16) significantly reduced HOMA‐IR, but produced no significant effect on other parameters of glucose metabolism and lipid profiles. In the pooled data analysis, we found high heterogeneity in the analysis of weight change. This high heterogeneity was reversed after the removal of Kunduraci and Ozbek (2020). We analyzed this study and found that although the participants included in this study were all overweight or obese, the BMI of the TRE group was higher than that of the control group, which may be the reason for heterogeneity. (The parameters of glucose and lipid metabolism and fat mass were consistent at baseline.) However, since the results of the weight change remained unchanged after the removal of this article and the results were the same as those of Kunduraci and Ozbek (2020), we believe that the results of the pooled data analysis were stable.

Therefore, TRE is a novel therapeutic strategy for weight control. Preliminary studies have demonstrated its feasibility and benefits in weight control and metabolism (Gabel et al., 2018; Moro et al., 2016; Ravussin et al., 2019; Stote et al., 2007). Kesztyüs et al. (2019) reported that TRE was widely accepted by participants, and 86% of them achieved their weight loss goal during a 3‐month study period. Several studies have reported other effects of TRE, in addition to body weight and metabolism. It was found that 4‐ and 6‐h TRE may improve some aspects of cardiometabolic health (Cienfuegos et al., 2020). TRE could also be beneficial in reducing inflammation and may have a protective effect on some components of the immune system (Moro et al., 2020). However, the results regarding the effects of TRE on sleep improvement have been inconsistent. In a 10‐h TRE study by Gill and Panda (2015), overweight participants experienced improved sleep quality after 16 weeks of intervention. Another study reported that a 10‐h TRF improved morning restfulness but had no effect on sleep quality after 12 weeks in participants with metabolic syndrome (Wilkinson et al., 2020). In contrast, Cienfuegos et al. (2022) suggested that 4‐ and 6‐h TRF have no effect on sleep quality, duration, insomnia severity, or risk of obstructive sleep apnea.

In this meta‐analysis, TRE (8/16) achieved significant weight loss and fat mass reduction in overweight and obese adults, but exerted no effect on the lean mass of the body. However, after subgroup analysis, we found that TRE (8/16) reduced body weight, fat mass, and lean mass simultaneously for people over 40 years of age compared to the control group, but it had no significant effect on body weight, fat mass, and lean mass in the <40 years old group. The potential health and metabolic benefits of weight loss are associated with minimal reduction in lean mass (Cava et al., 2017). When the reduction in fat‐free mass exceeds fat mass (i.e., increased risk of sarcopenia) during weight loss, these benefits are likely to be compromised. However, in this meta‐analysis, we found that although TRE (8/16) led to lean mass loss (with a reduction of 0.88 kg, *p* = .002), the weight reduction led to a greater fat mass loss (with a reduction of 1.17 kg, *p* = .0001) in participants ≥40 years old. Moon et al. (2020) arrived at the same conclusion: They believed that aging is associated with gradual loss of fat‐free mass and gain of excess body fat. While TRE can reduce this ratio, it might be a good therapeutic strategy for obesity in elderly patients.

Previous studies have shown that exercise can increase lean mass (Peterson et al., 2011). We found that one of the eight studies included regularly physically active participants. Therefore, subgroup analysis was performed. In addition to body weight reduction, TRE (8/16) reduced fat and lean mass in nonregularly physically active participants. However, there was only one study (Isenmann et al., 2021) in the regular physically active subgroup, and no pooled analysis was possible. In this study (Isenmann et al., 2021), it was shown that there were no significant changes in body weight, fat mass, and lean mass among regularly physically active participants compared to the control group. Previous studies (Brady et al., 2021; Moro et al., 2020; Tinsley et al., 2019) which included physically active participants (i.e., elite cyclists, long‐distance runners) demonstrated weight reduction with TRE (8/16) intervention. However, in these studies, the participants were neither overweight nor obese. Therefore, more RCTs are needed to assess the impact of TRE (8/16) on weight control in overweight or obese regular physically active populations.

In this meta‐analysis, pooled data showed that eTRE reduced body weight, but dTRE did not. A similar conclusion was reached in a rodent study (Regmi et al., 2021), which showed that eTRE reduced weight and fat mass more than dTRE. Xie et al. (2022) reported that the eTRE group showed a reduction in body mass, percentage body fat, and fat mass, but not in the dTRE group. This may be associated with the circadian rhythm. Disturbances in the daily rhythms of secreted substances are associated with obesity and metabolic health (Engin, 2017; Kaneko et al., 2009; Szewczyk‐Golec et al., 2015), while diet influences these (Chowdhury, Richardson, Holman, et al., 2016; Chowdhury, Richardson, Tsintzas, et al., 2016; Gavrila et al., 2003; Maeda et al., 2013). As suggested by the early eTRE proposal, alignment of the food intake period with the central circadian “clock” can lead to improvements in the regulation of lipid, protein, and carbohydrate (Reinke & Asher, 2016).

In the nonenergy‐restricted subgroup, the weight was lower than that before the TRE (8/16) intervention; however, there was no significant weight loss compared to the control group. Interestingly, in the energy‐restricted subgroup, the TRE group had more weight loss than the control group under the same energy‐restricted proportion in both groups. It is possible that TRE and energy restriction have a stack effect on weight control; however, the mechanism needs further study. In addition, TRE (8/16) intervention reduced fat mass without affecting lean mass in both the energy‐restricted and nonenergy‐restricted subgroups. Therefore, TRE could still be a healthy eating regimen.

The pooled data showed that waist circumference decreased in the TRE (8/16) group, whereas the waist–hip ratio and visceral fat did not change significantly. This indicates that waist circumference alone does not reduce visceral fat. Previous studies have shown that the best predictor of visceral fat is the waist‐to‐hip ratio (Ibrahim & Ahsan, 2019). In addition, TRE (8/16) reduced fat mass, but did not affect lipid profiles in overweight and obese participants. This may be because the TRE (8/16) reduction was due to subcutaneous fat, but not visceral fat. The decrease in visceral fat is related to changes in the lipid profiles (Tchernof & Després, 2013). Therefore, TRE (8/16) may have no effect on the lipid profiles.

Time‐restricted eating (8/16) intervention did significantly reduce HOMA‐IR, but there were no significant changes in blood glucose, insulin, and HbA1c levels. IR is now used as a screening indicator for the primary prevention of DM. IR commonly coexists with obesity (Tang et al., 2015). There is a strong positive correlation between the volume of adipose tissue and the level of insulin resistance (Banerji et al., 1997). The basic reason for IR in obesity lies in the products released from adipose tissue which include free fatty acids and inflammatory mediators such as TNF alpha. These products contribute to both peripheral and hepatic IR. Increased insulin sensitivity or remission of T2DM has been reported in patients who have undergone bariatric surgery (Rao et al., 2012). Therefore, HOMA‐IR reduction may be associated with fat mass reduction and weight loss.

Regarding blood pressure, the pooled data demonstrated that there was no significant difference between the TRE (8/16) and control groups. This is inconsistent with the results of previous meta‐analyses (Chen et al., 2021; Moon et al., 2020), which concluded that TRE lowered blood pressure. This may be related to many factors, such as the included population, intervention duration, and fasting window, which were inconsistent in these studies. Nevertheless, we believe that the most important reason was the fact that most studies were performed over a short period, which made it difficult to evaluate the long‐term benefits of TRE on cardiovascular health.

In addition, because TRE does not limit the quality or quantity of food, participants show higher adaptability to this eating habit (Adafer et al., 2020; Wilkinson et al., 2020). In this meta‐analysis, two studies (Isenmann et al., 2021; Liu et al., 2022) reported that the TRE (8/16) group had relatively high adherence rates (84% and 98.4%, respectively). Not only adherence, but also long‐term adherence is the most important factor for a dietary habit to bring tangible benefits to overall health (Middleton et al., 2013). In a trial conducted by Wilkinson et al. (2020), 63% of participants maintained some amount of TRE for 16 months after the end of the 12‐week intervention. These findings suggest that TRE may be a sustainable dietary intervention.

No serious adverse effects were reported in any of the included studies. Kotarsky et al. (2021) reported that one participant had morning headache. Liu et al. (2022) reported occurrences of mild adverse events such as fatigue, dizziness, headache, decreased appetite, upper abdominal pain, dyspepsia, and constipation during the 12‐month follow‐up, and there was no significant difference between the TRE (8/16) and control groups.

Several studies have suggested that circadian rhythms and gut microbiota play a role in the metabolic regulation of TRE. In mammalian studies, the intrinsic circadian system is a complicated feedback network that maintains and regulates proper rhythms in metabolic pathways required for homeostasis (Green et al., 2008; Takahashi et al., 2008). The desynchronization of normal circadian rhythms is associated with various pathological conditions such as obesity and related metabolic disorders (Challet, 2015). In animal research, TRE can alter the hepatic expression level of core circadian proteins and mRNAs, resulting in changes in circadian rhythmicity (Patel, 2016). In a clinical study, it was also found that TRE might enhance the daily rhythms of human clock genes (Xie et al., 2022) to achieve the role of metabolic regulation. Gut microbiota has been reported to be another possible mechanism. Obesity and metabolic disorders are related to the type and diversity of the gut microbiota. An animal study reported that *Lactobacillus* and *Lactococcus* species, which are thought to be obesogenic (Joyce et al., 2014; Million et al., 2012) had significant reduction by TRE intervention (Zarrinpar et al., 2014). Gut microbiota diversity, particularly α‐diversity reduction, is thought to play a significant role in host metabolic diseases and obesity (Lozupone et al., 2012). In clinical studies, pTregs and α diversity of the gut microbiota increased with TRE intervention, and these have been reported to contribute to the beneficial effects of reducing the risk of metabolic diseases (Xie et al., 2022; Zeb et al., 2020). However, the exact mechanism underlying the effects of TRE on obesity and metabolism remains unclear.

This systematic review and meta‐analysis had some limitations. First, since TRE is a new dietary strategy developed in recent years, the number of articles was small. Some outcomes have been evaluated in a limited number of studies. Second, most included studies had a small sample size, partly giving rise to high heterogeneity among studies in the meta‐analyses. In addition, most studies were conducted over a short period, so it was difficult to determine the long‐term benefits and safety of TRE. Finally, owing to the lack of data, it is challenging to analyze the blood parameters and gut microbiota associated with obesity and metabolic dysfunction.

In conclusion, TRE (8/16), especially eTRE, or in combination with caloric intake restriction, is a potential therapeutic strategy for weight control in overweight and obese adults. TRE (8/16) also reduced HOMA‐IR; therefore, it may have a positive effect on glucose metabolism.

## CONFLICT OF INTEREST

The authors declare no conflicts of interest.

## ETHICAL APPROVAL

This review used only published data sources. An ethical review by the Research Ethics Committee was not required.

## Supporting information


Appendix S1.

Appendix S2.

## Data Availability

This review used only published data sources. Therefore, no raw data were available.
