# Supplementary material for: Is time‐restricted eating (8/16) beneficial for body weight and metabolism of obese and overweight adults? A systematic review and meta‐analysis of randomized controlled trials
Source: Food Sci Nutr. 2022 Dec 19;11(3):1187–200. doi: 10.1002/fsn3.3194 (PMC10002957; doi:10.1002/fsn3.3194)
Supplement: Supplementary file 1 — Appendix S1. [file FSN3-11-1187-s002.docx]

EBM Reviews - Cochrane Central Register of Controlled Trials <April 2022>

((time restricted feeding or time restricted eating or time restricted meal or time restricted feedings or time restricted diet or time restricted fasting or Intermittent Fasting) and (Overweight or Adiposity or Body Mass Index or Obesity or body weight or weight loss or weight gain or waist circumference or Body Size or Body Fat Distribution or Body composition or Insulin Resistance or blood pressure or Gastrointestinal Microbiome or Diabetes Mellitus or Cholesterol or Triglycerides or non-alcoholic fatty liver disease or Glucose Metabolism Disorders or body mass or body fat or body lean mass or plasma glucose or Fasting glucose or serum lipids or Inflammatory markers or total cholesterol or Quetelet Index or Quetelet's Index or Quetelets Index or Weight Losses or Weight Reduction or Weight Reductions or Waist Circumferences or Body Sizes or Body Fat Patterning or Body Compositions or Insulin Sensitivity or Diastolic Pressure or Pulse Pressure or Systolic Pressure or Epicholesterol or Triacylglycerols or Triacylglycerol or Triglyceride or High-density lipoprotein or Low Density Lipoprotein or Nonalcoholic Steatohepatitis or Nonalcoholic Steatohepatitides or NAFLD) and (Randomized Controlled Trial or controlled clinical trial or random allocation or double-blind or single-blind or placebo or randomly or randomized or RCT)).af. 255

Embase <1974 to 2022 May 06>

((time restricted feeding or time restricted eating or time restricted meal or time restricted feedings or time restricted diet or time restricted fasting or Intermittent Fasting) and (Overweight or Adiposity or Body Mass Index or Obesity or body weight or weight loss or weight gain or waist circumference or Body Size or Body Fat Distribution or Body composition or Insulin Resistance or blood pressure or Gastrointestinal Microbiome or Diabetes Mellitus or Cholesterol or Triglycerides or non-alcoholic fatty liver disease or Glucose Metabolism Disorders or body mass or body fat or body lean mass or plasma glucose or Fasting glucose or serum lipids or Inflammatory markers or total cholesterol or Quetelet Index or Quetelet's Index or Quetelets Index or Weight Losses or Weight Reduction or Weight Reductions or Waist Circumferences or Body Sizes or Body Fat Patterning or Body Compositions or Insulin Sensitivity or Diastolic Pressure or Pulse Pressure or Systolic Pressure or Epicholesterol or Triacylglycerols or Triacylglycerol or Triglyceride or High-density lipoprotein or Low Density Lipoprotein or Nonalcoholic Steatohepatitis or Nonalcoholic Steatohepatitides or NAFLD) and (Randomized Controlled Trial or controlled clinical trial or random allocation or double-blind or single-blind or placebo or randomly or randomized or RCT)).af. 321

Ovid MEDLINE(R) <1946 to April Week 5 2022>

((time restricted feeding or time restricted eating or time restricted meal or time restricted feedings or time restricted diet or time restricted fasting or Intermittent Fasting) and (Overweight or Adiposity or Body Mass Index or Obesity or body weight or weight loss or weight gain or waist circumference or Body Size or Body Fat Distribution or Body composition or Insulin Resistance or blood pressure or Gastrointestinal Microbiome or Diabetes Mellitus or Cholesterol or Triglycerides or non-alcoholic fatty liver disease or Glucose Metabolism Disorders or body mass or body fat or body lean mass or plasma glucose or Fasting glucose or serum lipids or Inflammatory markers or total cholesterol or Quetelet Index or Quetelet's Index or Quetelets Index or Weight Losses or Weight Reduction or Weight Reductions or Waist Circumferences or Body Sizes or Body Fat Patterning or Body Compositions or Insulin Sensitivity or Diastolic Pressure or Pulse Pressure or Systolic Pressure or Epicholesterol or Triacylglycerols or Triacylglycerol or Triglyceride or High-density lipoprotein or Low Density Lipoprotein or Nonalcoholic Steatohepatitis or Nonalcoholic Steatohepatitides or NAFLD) and (Randomized Controlled Trial or controlled clinical trial or random allocation or double-blind or single-blind or placebo or randomly or randomized or RCT)).af. 176

Pubmed

(((Overweight or Adiposity or Body Mass Index or Obesity or body weight or weight loss or weight gain or waist circumference or Body Size or Body Fat Distribution or Body composition or Insulin Resistance or blood pressure or Gastrointestinal Microbiome or Diabetes Mellitus or Cholesterol or Triglycerides or non-alcoholic fatty liver disease or Glucose Metabolism Disorders[MeSH Terms]) OR (Obes*[Title/Abstract] OR Overweight*[Title/Abstract] OR Over weight*[Title/Abstract] OR Adiposit*[Title/Abstract] OR body mass[Title/Abstract] OR body fat[Title/Abstract] OR body lean mass[Title/Abstract] OR plasma glucose[Title/Abstract] OR Fasting glucose[Title/Abstract] OR serum lipids[Title/Abstract] OR Inflammatory markers[Title/Abstract] OR total cholesterol[Title/Abstract] OR Quetelet Index[Title/Abstract] OR Quetelet's Index[Title/Abstract] OR Quetelets Index[Title/Abstract] OR Weight Losses[Title/Abstract] OR Weight Reduction[Title/Abstract] OR Weight Reductions[Title/Abstract] OR Waist Circumferences[Title/Abstract] OR Body Sizes[Title/Abstract] OR Body Fat Patterning[Title/Abstract] OR Body Compositions[Title/Abstract] OR Insulin Sensitivity[Title/Abstract] OR Diastolic Pressure[Title/Abstract] OR Pulse Pressure[Title/Abstract] OR Systolic Pressure[Title/Abstract] OR Epicholesterol[Title/Abstract] OR Triacylglycerols[Title/Abstract] OR Triacylglycerol[Title/Abstract] OR Triglyceride[Title/Abstract] OR High-density lipoprotein[Title/Abstract] OR Low Density Lipoprotein[Title/Abstract] OR Nonalcoholic Steatohepatitis[Title/Abstract] OR NAFLD[Title/Abstract])) AND ((fasting[MeSH Terms]) OR (time restricted feeding[Title/Abstract] OR time restricted eating[Title/Abstract] OR time restricted meal[Title/Abstract] OR time restricted feedings[Title/Abstract] OR time restricted diet[Title/Abstract] OR time restricted fasting[Title/Abstract] OR Intermittent Fasting[Title/Abstract] OR time restricted*[Title/Abstract]))) AND ((Randomized Controlled Trial[MeSH Terms]) OR (controlled clinical trial[Title/Abstract] OR random allocation[Title/Abstract] OR double-blind[Title/Abstract] OR single-blind[Title/Abstract] OR placebo[Title/Abstract] OR randomly[Title/Abstract] OR randomized[Title/Abstract] OR RCT[Title/Abstract] OR clinical trial*[Title/Abstract] OR trial*[Title/Abstract] OR random*[Title/Abstract])) 3,373
