# Supplementary material for: Is time‐restricted eating (8/16) beneficial for body weight and metabolism of obese and overweight adults? A systematic review and meta‐analysis of randomized controlled trials
Source: Food Sci Nutr. 2022 Dec 19;11(3):1187–200. doi: 10.1002/fsn3.3194 (PMC10002957; doi:10.1002/fsn3.3194)
Supplement: Supplementary file 2 — Appendix S2. [file FSN3-11-1187-s001.doc]

Table S1 Risk of bias summary: risk of bias item for each included RCT for meta-analysis according to Cochrane Risk-of-Bias Tool.

| Studies | Random sequence generation (selection bias) | Allocation concealment (selection bias) | Blinding of participants and personnel (performance bias) | Blinding of outcome assessment (detection bias) | Incomplete outcome data (attrition bias) | Selective reporting (reporting bias) | Other bias |
| --- | --- | --- | --- | --- | --- | --- | --- |
| Amodio, D. et al. 2016 | Unclear risk. Not reported. | Unclear risk. Not reported. | Unclear risk. Not reported. | Unclear risk. Not reported. | Low risk. Low dropout rate. | Unclear risk. Not reported. | Low risk. Received no external funding. |
| Chow, L. S. et al. 2020 | Low risk. Using the SAS pseudorandom number generator procedure | Low risk. Central allocation, web-based randomization. | Unclear risk. Not reported. | Unclear risk. Not reported. | Low risk. Low dropout rate. And the number and reasons of dropout were reported | Low risk. Prespecified outcomes available on a clinical trial database and all reported in publication. | Low risk. Funding source was obtained from non-profitable organizations. |
| Domaszewski, P. et al. 2020 | Unclear risk. Not reported. | Unclear risk. Not reported. | Unclear risk. Not reported. | Unclear risk. Not reported. | Unclear risk.The number and reasons of dropout were not reported | Unclear risk. Not reported. | Low risk. Received no external funding. |
| Isenmann, E. et al. 2021 | Unclear risk. Not reported. | Unclear risk. Not reported. | Unclear risk. Not reported. | Unclear risk. Not reported. | Low risk. Low dropout rate. And the number and reasons of dropout were reported | Unclear risk. Not reported. | Low risk. Received no external funding. |
| Kotarsky, C. J. et al. 2021 | Unclear risk. Not reported. | Unclear risk. Not reported. | Unclear risk. Not reported. | Unclear risk. Not reported. | Low risk. Low dropout rate. And the number and reasons of dropout were reported | Low risk. Prespecified outcomes available on a clinical trial database and all reported in publication | Low risk. Funding source was obtained from non-profitable organizations. |
| Kunduraci, Y. E. et al. 2020 | Low risk. using computerized random number generation. | Low risk. Central allocation, web-based randomization. | Unclear risk. Not reported. | Unclear risk. Not reported. | Low risk. Low dropout rate. And the number and reasons of dropout were reported. | Low risk. Prespecified outcomes available on a clinical trial database and all reported in publication. | Low risk. Received no external funding. |
| Liu, D. et al. 2022 | Unclear risk. Not reported. | Unclear risk. Not reported. | Low risk.Observers (trial personnel) were unaware of group assignments. | Low risk. Observers (trial personnel) were unaware of group assignments. | Low risk. Low dropout rate. And the number and reasons of dropout were reported. | Low risk. Prespecified outcomes available on a clinical trial database and all reported in publication. | Low risk. Funding source was obtained from non-profitable organizations. |
| Lowe, D. A. et al. 2020 | Low risk. Computer generated list. | Low risk. Central allocation, web-based randomization. | Unclear risk. Not reported. | Unclear risk. Not reported. | Low risk. Low dropout rate. And the number and reasons of dropout were reported. | Low risk. Prespecified outcomes available on a clinical trial database and all reported in publication. | Low risk. There was industry involvement but no high risk of bias feature encountered. |


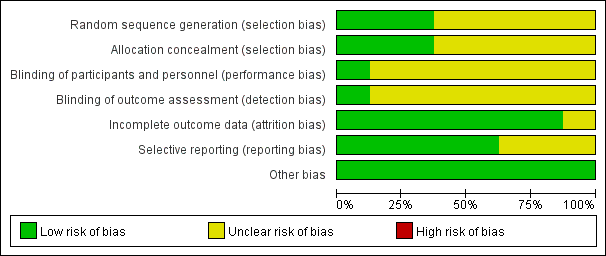


Figure S1. Risk of bias graph: each risk of bias item is presented as percentages across all included RCTs.


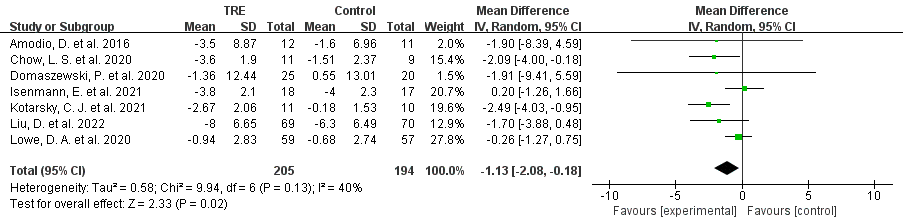


Figure S2. Effect of TRE versus controls on change of body weight in individuals and the analysis was based on results obtained from sensitivity analysis using leave-one-out approach with heterogeneous studies identified.

A


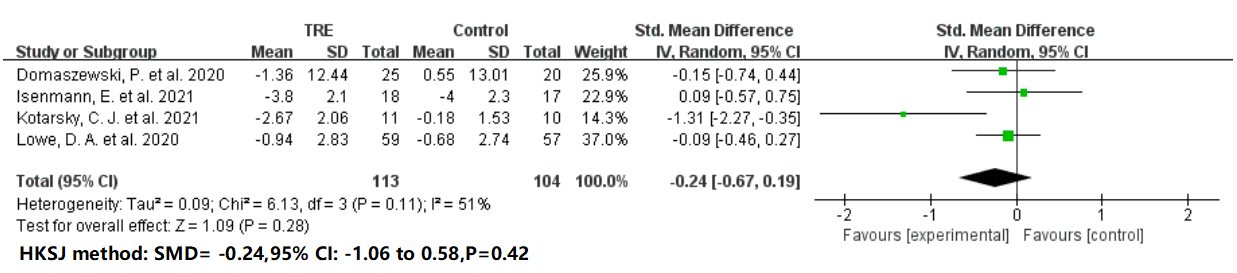


B


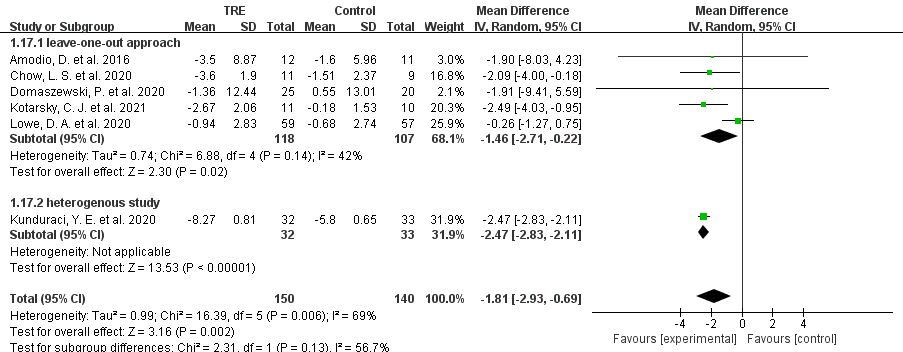


C


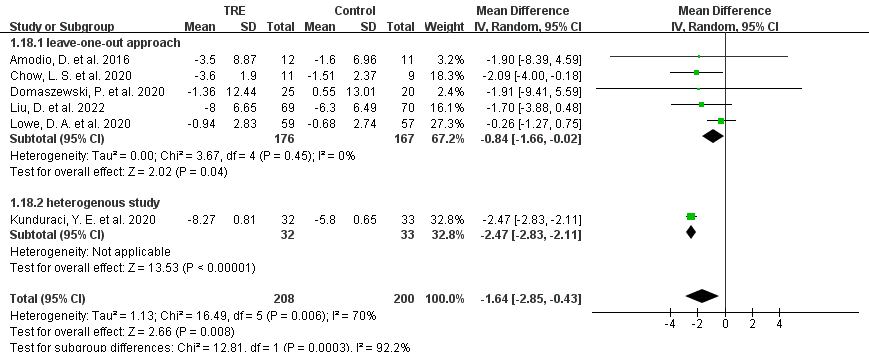


D


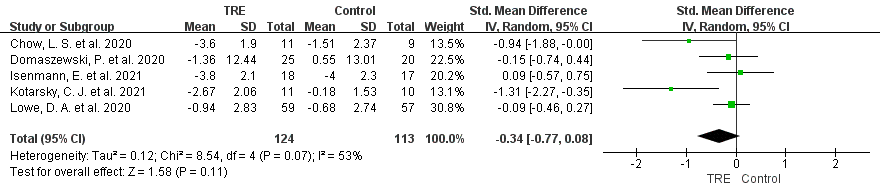

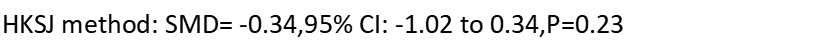


Figure S3. Sensitivity analysis and HKSJ analysis for the data pooled with high heterogeneity. A. Forest plot for body weight loss of dTRE vs. Controls using HKSJ method. CI confdence interval, HKSJ Hartung-Knapp-Sidik-Jonkman, SMD standardized mean diference. B. The body weight loss in the≥ 40 old years group, by removing a heterogeneous study by Kunduraci, Y. E. et al. (2020) based on leave-one-out approach. C. The body weight loss in the non-regularly physically active group, by removing a heterogeneous study by Kunduraci, Y. E. et al. (2020) based on leave-one-out approach. D. Forest plot for body weight loss of TRE vs. Controls in non-energy restriction group using HKSJ method.

A


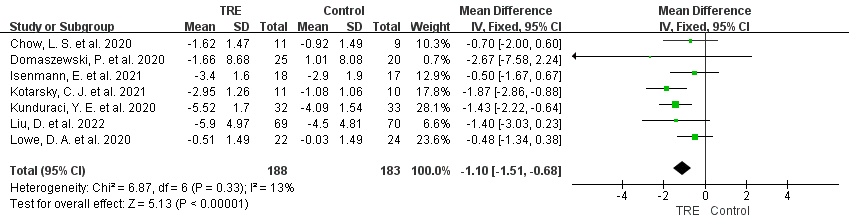


B


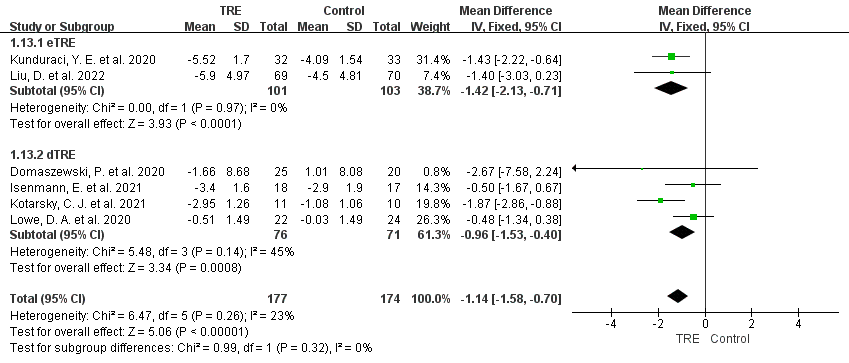


C


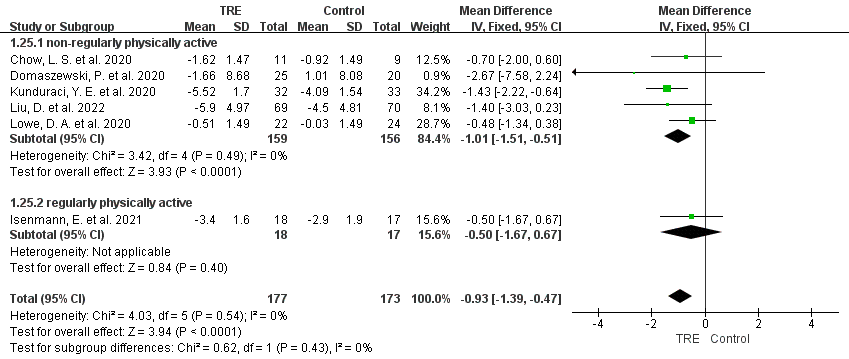


D


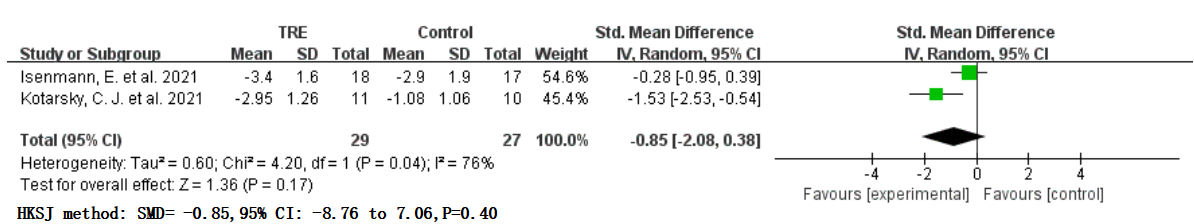


E


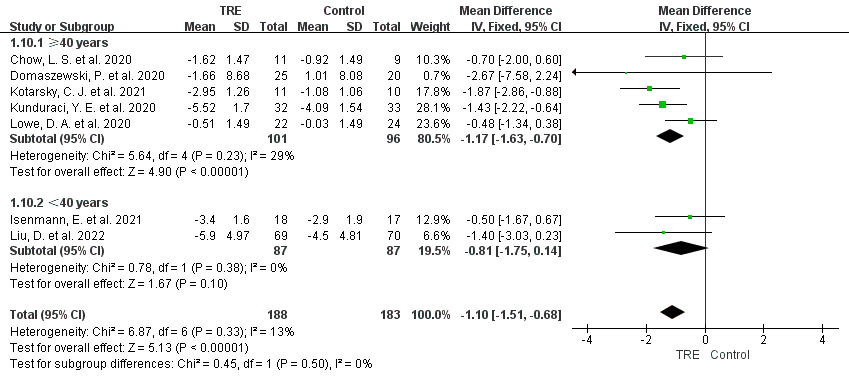


F


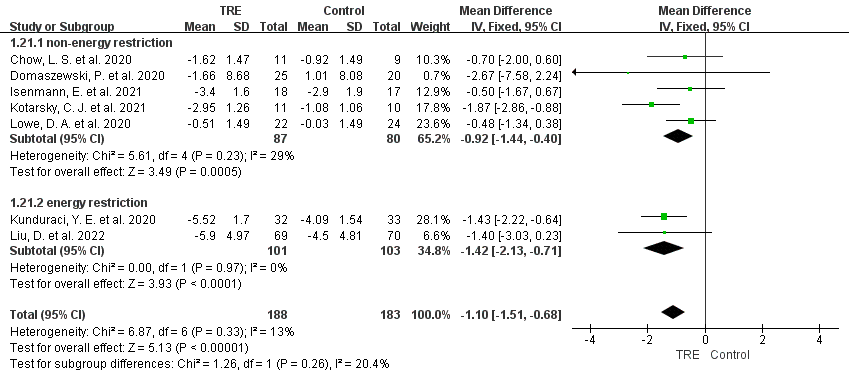


G


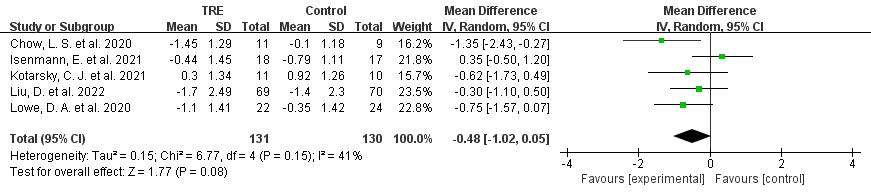


H
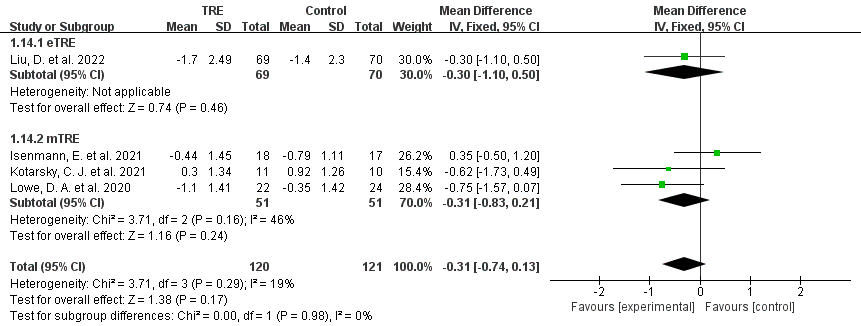


I


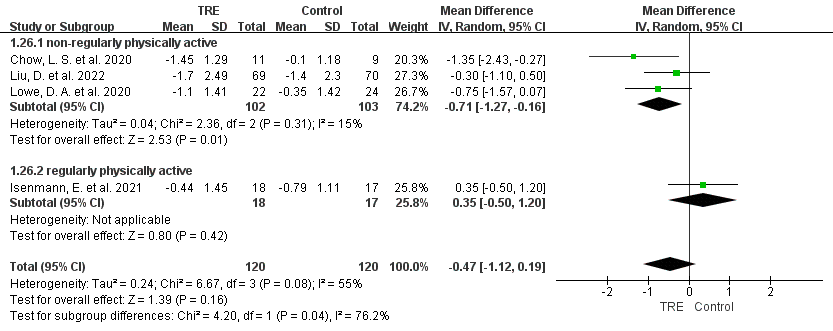


J


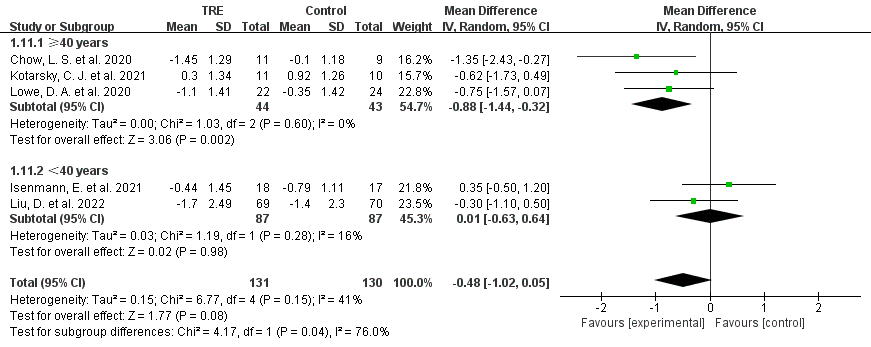


K


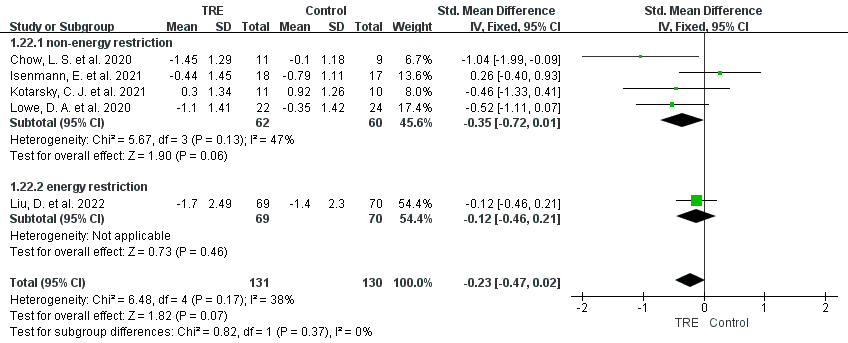


L


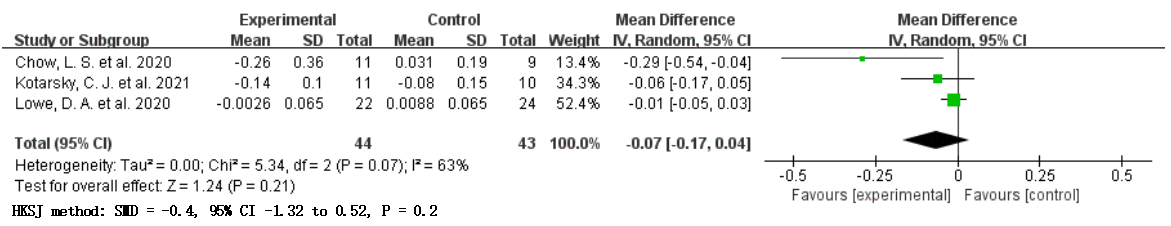


Figure S4 Effect of TRE versus control on changes of body composition. A. Effect of TRE versus control on changes of body fat mass. B. The effect on the fat mass loss of TRE vs. controls following subgroup assignment based on fasting windows. C. The effect on the fat mass loss of TRE vs. controls following subgroup assignment based on different amount of exercise. D. Forest plot for body fat mass loss of regularly physically active group vs. Controls. CI confdence interval, HKSJ Hartung-Knapp-Sidik-Jonkman, SMD standardized mean diference. E. The effect on the fat mass loss of TRE vs. controls following subgroup assignment based on ages. F. The effect on the fat mass loss of TRE vs. controls following subgroup assignment based on limit energy intake or not. G. Effect of TRE versus control on changes of body lean mass. H. The effect on the lean mass loss of TRE vs. controls following subgroup assignment based on fasting windows. I. The effect on the lean mass loss of TRE vs. controls following subgroup assignment based on different exercise. J. The effect on the lean mass loss of TRE vs. controls following subgroup assignment based on ages. K. The effect on the lean mass loss of TRE vs. controls following subgroup assignment based on limit energy intake or not. L. The effect of TRE versus control on changes of visceral fat mass.

A


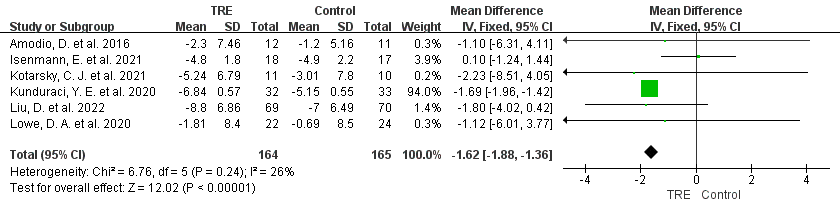


B


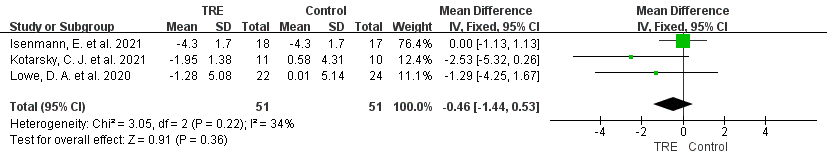


Figure S5 Effect of TRE versus control on changes of waist circumference and hip circumference. A. Effect of TRE versus control on changes of waist circumference. B. Effect of TRE versus control on changes of hip circumference.

A


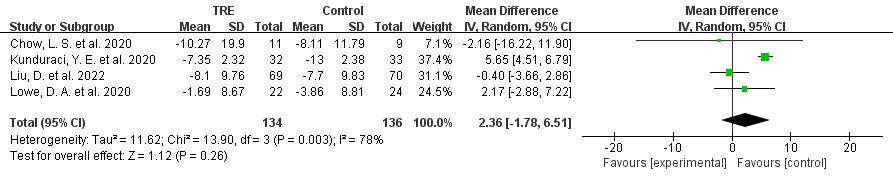


B


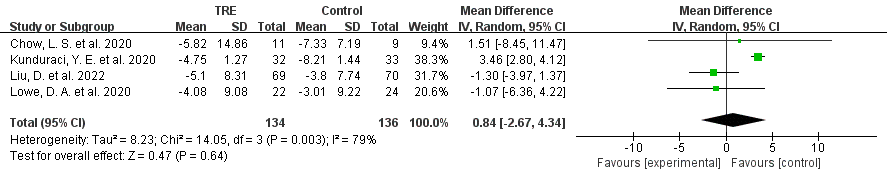


Figure S6 Effect of TRE versus control on changes of blood pressure. A. Effect of TRE versus control on changes of systolic blood pressure. B. Effect of TRE versus control on changes of diastolic blood pressure.

A


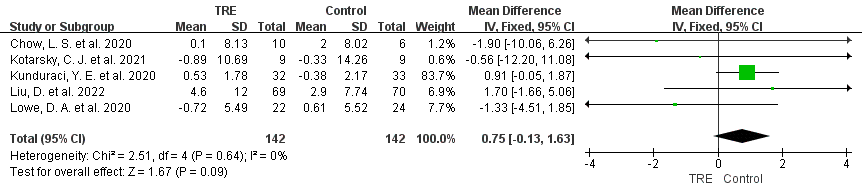


B


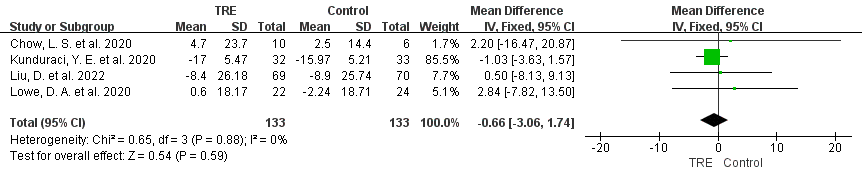


C


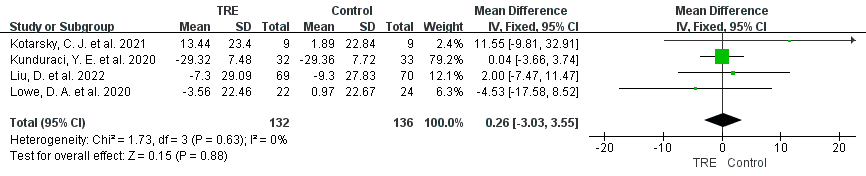


D


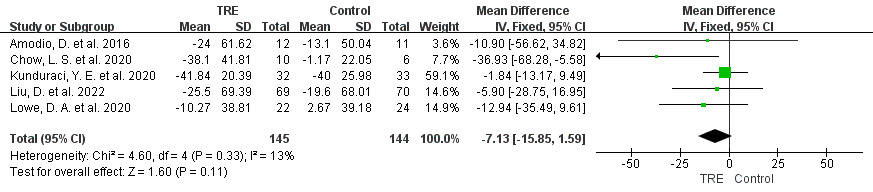


Figure S7 Effect of TRE versus control on changes of lipid profiles. A. Effect of TRE versus control on changes of high-density lipoprotein cholesterol (HDL-C). B. Effect of TRE versus control on changes of low-density lipoprotein cholesterol (LDL-C). C. Effect of TRE versus control on changes of total cholesterol (TC). D. Effect of TRE versus control on changes of triglycerides(TG).

A


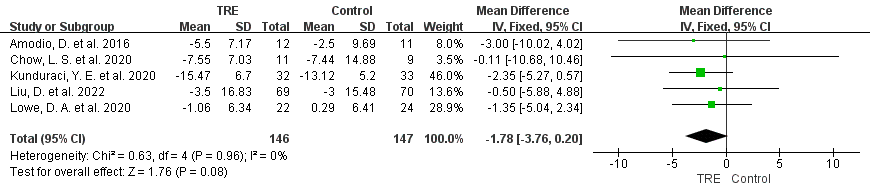


B


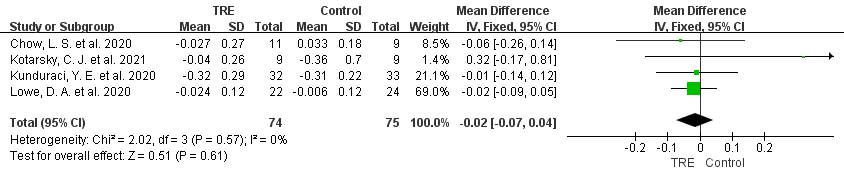


C


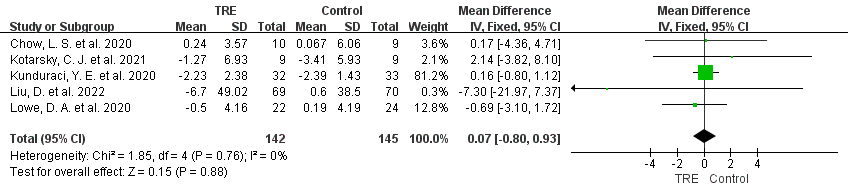


D


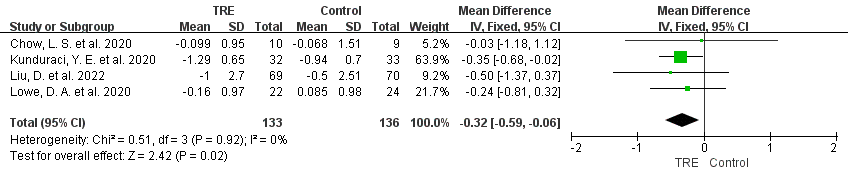


Figure S8 Effect of TRE versus control on changes of glucose metabolism. A. Effect of TRE versus control on changes of blood glucose. B. Effect of TRE versus control on changes of hemoglobin A1c (HbA1c). C. Effect of TRE versus control on changes of insulin. D. Effect of TRE versus control on changes of homeostatic model assessment of insulin resistance (HOMA-IR).
